# *FGFR3* Mutations in Urothelial Carcinoma: A Single-Center Study Using Next-Generation Sequencing

**DOI:** 10.3390/jcm13051305

**Published:** 2024-02-25

**Authors:** Seong Hyeon Yu, Sung sun Kim, Shinseung Kim, Hyungki Lee, Taek Won Kang

**Affiliations:** 1 Department of Urology, Chonnam National University Medical School, Chonnam National University Hospital, Gwangju 61469, Republic of Korea; domer12@hanmail.net; 2 Department of Pathology, Chonnam National University Medical School, Chonnam National University Hospital, Gwangju 61469, Republic of Korea; kimsspathology@jnu.ac.kr; 3 MediCloud Corporation, Hwasun 58128, Republic of Korea; sskim@medicloudgroup.com (S.K.); hklee@medicloudgroup.com (H.L.)

**Keywords:** next-generation sequencing, fibroblast growth factor receptor, transitional cell carcinoma

## Abstract

**Background:** Mutations of fibroblast growth factor receptor 3 (*FGFR3*) are associated with urothelial carcinoma (UC) oncogenesis and are considered an important therapeutic target. Therefore, we evaluated the *FGFR3* mutation rate and its clinical significance in urothelial carcinoma (UC) using next-generation sequencing. **Methods:** A total of 123 patients with UC who were treated at Chonnam National University Hospital (Gwang-ju, Korea) from January 2018 to December 2020 were enrolled. We performed NGS using the Oncomine panel with tumor specimens and blood samples corresponding to each specimen. We analyzed the *FGFR3* mutation results according to the type of UC and the effects on early recurrence and progression. **Results:** The mean age of the patients was 71.39 ± 9.33 years, and 103 patients (83.7%) were male. Overall, the *FGFR3* mutation rate was 30.1% (37 patients). The *FGFR3* mutation rate was the highest in the non-muscle-invasive bladder cancer (NMIBC) group (45.1%), followed by the muscle-invasive bladder cancer (22.7%) and upper tract UC (UTUC) (14.3%) groups. Patients with *FGFR3* mutations had a significantly lower disease stage (*p* = 0.019) but a high-risk of NMIBC (*p* < 0.001). **Conclusions:** Our results revealed that *FGFR3* mutations were more prevalent in patients with NMIBC and lower stage UC and associated with a high-risk of NMIBC. Large multicenter studies are needed to clarify the clinical significance of *FGFR3* mutations in UC.

## 1. Introduction

Urothelial carcinoma (UC) can be found in the upper and lower urinary tracts. The most important risk factor for developing this type of cancer is tobacco smoking, which accounts for 50% of cases [1]. The majority of UCs are bladder cancer—the 10th most commonly diagnosed cancer worldwide [2]—which can be divided into two main categories, i.e., non-muscle-invasive bladder cancer (NMIBC) and muscle-invasive bladder cancer (MIBC). Approximately 75% of patients with bladder cancer are diagnosed as having NMIBC, with a high cancer-specific survival rate, whereas MIBC, which is more likely to spread to lymph nodes or other organs, has a poor prognosis [3,4]. Upper tract UC (UTUC), which is uncommon and accounts for only 5–10% of cases of UC, usually exhibits an aggressive behavior [5]. With recent developments in medical oncology, although platinum-based chemotherapy has been the cornerstone of therapeutic strategies for patients with advanced or metastatic UC, the survival benefits remain dismal, with a median overall survival of approximately 15 months [6].

In recent years, the therapeutic scenario has evolved with the development and approval of several immune checkpoint inhibitors [7]. In addition, the rapid development of clinical genetic testing techniques such as next-generation sequencing (NGS) has facilitated a better understanding of the molecular landscape, which enables disease risk assessment and informative biomarker identification and leads to the development of novel selective genomic-targeting therapeutics for the treatment of UC patients. Therefore, the use of NGS is rapidly gaining popularity in the clinical practice of UC, similar to non-small cell lung, breast and colorectal cancers [8].

Among potentially targetable genomic alterations, mutations of fibroblast growth factor receptor 3 (*FGFR3*), which are one of the most common somatic mutations in UC, are associated with UC oncogenesis and are considered an important therapeutic target [6,9]. As mentioned above, NGS has facilitated the identification of *FGFR3* mutations in a wide range of cancers as well as UC [8]. As a result, *FGFR3* mutation tests are increasingly performed, leading to the development and commercialization of *FGFR3*-targeting therapeutics (e.g., erdafitinib) in clinical practice [9]. *FGFR3* mutations are generally associated with low-grade and low-stage UC and favorable disease-specific survival [10,11]. However, there is also evidence suggesting that *FGFR3* mutations are associated with a less favorable prognosis in advanced UC [12]. Furthermore, compared with Western countries, there are limited data on *FGFR3* mutations in Asian countries, especially in South Korea. Hence, in the present study, we aimed to investigate the *FGFR3* mutation rate and its clinical significance in UC using NGS.

## 2. Materials and Methods

### 2.1. Study Population and Data Collection

The present study screened patients who visited Chonnam National University Hospital (Gwang-ju, Korea) for the treatment of UC from January 2018 to December 2020. All patients were diagnosed by histologic confirmation through transurethral resection of bladder tumor (TURBT), radical nephroureterectomy (RNU) or tumor biopsy (only for metastatic diseases). Urothelial tumors were staged according to the 2017 TNM classification of UC [13]. The histologic grading of urothelial tumors was performed using the 2004/2016 World Health Organization (WHO) grading system [14]. In addition, patients diagnosed with NMIBC were stratified into 4 groups according to the European Association of Urology prognostic factor risk groups [3]. UC specimens for NGS analysis were obtained at the initial TURBT, RNU or tumor biopsy, and blood samples corresponding to each specimen were collected when the patients visited the outpatient department as scheduled. Patients were excluded for any of the following reasons: UC specimens not suitable for NGS analysis, histologic diagnosis other than pure transitional cell carcinoma, inability to collect blood samples or obtain informed consent or follow-up loss. A total of 123 patients were included in the final analysis based on these exclusion criteria.

A detailed medical history, including age, body mass index (BMI), sex, diabetes mellitus, hypertension, clinical stage, histologic grade, risk stratification (only if NMIBC), bladder tumor recurrence and disease progression were obtained from the patients’ medical records. Patients were monitored by cystoscopy regularly every 3 to 6 months and computed tomography (CT) every 3 to 6 months according to the stage of the disease. Disease recurrence was defined as the cystoscopic detection of a new bladder tumor with histologic confirmation. Disease progression was defined as the worsening of clinical status with or without radiologic progression, consequently leading to a change in treatment.

### 2.2. NGS Analysis

An NGS analysis of *FGFR3* mutations in UC was performed on UC specimens (formalin-fixed paraffin-embedded tissue) obtained from the initial TURBT, RNU or tumor biopsy and blood samples corresponding to each specimen collected from patients. The slides of all bladder cancer specimens were reviewed by a histopathologic specialist. Genomic DNA was extracted from 123 tumor samples and their corresponding blood samples. Genomic DNA was extracted using the QIAGEN GeneRead DNA FFPE Kit (QIAGEN, Hilden, Germany) following the manufacturer’s instructions. DNA concentration was quantified using the Qubit™ ds DNA High-Sensitive Assay Kit (Thermo Fisher Scientific, Waltham, MA, USA) on the Qubit fluorometer. 

Sequencing was performed with the Oncomine Comprehensive Assay v3 (OCAv3) panel (Thermo Fisher Scientific, Waltham, MA, USA). A library was prepared using the OCAv3 kit (Thermo Fisher Scientific, Waltham, MA, USA) according to the manufacturer’s manual. Multiplex PCR was performed using 20 ng of DNA. The completed library was quantified with a High Sensitivity DNA Kit (Cat. 5067–4626) on a 4200 TapeStation system (Agilent, Santa Clara, CA, USA) and diluted to a final concentration of 14 pM. Diluted samples were subjected to template prep with Ion Chef XL equipment (Thermo Fisher Scientific, Waltham, MA, USA). Template-prepped samples were loaded into the Ion 530™ Chip Kit (Cat. A45850) and analyzed on the Ion S5 XL sequencing equipment. Alignment was performed using t-map (v5.10.1). Hg19 was used as the reference genome [15].

The sequenced data were processed using a series of steps. We aligned the sequenced files (FASTQ file) to the reference genome (human reference genome g1k v37) using BBmap (38.96), and sorting and indexing were performed using Samtools (samtools-1.3.1). Next, filtered alignments were further processed to improve the alignment quality, including local realignment around indels and base quality score recalibration using the Genome Analysis Toolkit (gatk-4.2.6.1). Base quality score recalibration was carried out to recalculate base quality scores for all sequenced reads based on known polymorphisms. The base and mapping quality scores were used to filter reads during variant calling, and the fine-tuning that occurs in this step is important to ensure only high-confidence variants are called [15].

Variant calling was performed in GATK-Mutect2. Mutect is a method developed for detecting the most likely somatic point mutations in NGS data using a Bayesian classifier approach. We used FilterMutectCalls of the Mutect2 pipeline for a variant filter that filters based on the probability of a somatic variant and optimizes the threshold of the “F score” by considering the average of sensitivity and precision. By performing this variant calling with tumor (bladder tissue) and normal (whole blood) samples (“tumor with matched normal” mode) on each sample, germ-line mutations were excluded, and we analyzed oncogene mutations among pure somatic mutations [15]. Among them, we extracted the *FGFR3* mutation data, and analyzed the *FGFR3* mutation results according to the type of UC and the effects on disease recurrence and progression within 1 year. 

### 2.3. Statistical Analysis

Statistical analysis was performed using STATA version 16.1 (StataCorp, College Station, TX, USA). Descriptive analysis was performed to assess patient demographics. Continuous variables are presented as means and standard deviations, and categorical variables are presented as frequencies (%). Student’s *t*-test, Wilcoxon rank test, one-way ANOVA and Kruskal–Wallis rank test for continuous variables and Pearson chi-square test and Fisher’s exact test for categorical variables were used to compare clinical characteristics according to the type of UC and presence of *FGFR3* mutations. Logistic regression was performed to evaluate the effects on disease recurrence and progression within 1 year. A *p* value of <0.05 was considered indicative of statistical significance.

### 2.4. Ethics Statement

The study protocol was reviewed and approved by the institutional review board of Chonnam National University Hospital (IRB-approved protocol: No. CNUH-2023-254). The study was performed in accordance with the principles of the Declaration of Helsinki and the Ethical Guidelines for Clinical Studies. 

## 3. Results

The characteristics of patients who were eligible for NGS analysis using UC specimens are summarized in Table 1. The patients’ mean age and BMI were 71.39 ± 9.33 years and 24.10 ± 3.53 kg/m^2^, respectively. A total of 103 (83.7%) patients were male. In addition, 70 (56.9%) patients had hypertension, and 29 (23.6%) patients had diabetes mellitus. In terms of the type of UC, 51 (41.5%), 44 (35.8%) and 28 (22.8%) patients had NMIBC, MIBC and UTUC, respectively. In terms of the disease stage, the number of patients in stages 0a, 1, 2, 3 and 4 were 48 (39.0%), 3 (2.4%), 8 (6.5%), 39 (31.7%) and 25 (20.3%), respectively. According to the European Association of Urology prognostic factor risk groups, among 51 patients with NMIBC, 14 (27.4%), 21 (41.2%) and 16 (31.4%) patients belonged to the low risk, intermediate risk and high-risk groups, respectively. Furthermore, 27 (39.7%) of 72 patients with MIBC and UTUC underwent radical surgery with a curative intent. During the follow-up period, 28 (54.9%) of 51 patients with NMIBC had disease recurrence, 26 (36.1%) of 72 patients with MIBC and UTUC had disease progression and 4 (14.3%) of 28 patients with UTUC had disease recurrence in the bladder (all within 1 year). Of the 123 patients with UC, 37 (30.1%) patients were presented with *FGFR3* mutations.

There were significant differences between the 3 groups (NMIBC, MIBC, UTUC) in the *FGFR3* mutation rate according to the type of UC (*p* = 0.007). Specifically, the *FGFR3* mutation rate was the highest in the NMIBC group (45.1%), followed by the MIBC (22.7%) and UTUC (14.3%) groups (Table 2). A comparison of clinical features according to the presence of *FGFR3* mutations revealed that the *FGFR3* mutation group was significantly associated with a lower disease stage (*p* = 0.021) and a high-risk of NMIBC (*p* < 0.001) (Table 3).

The predictive factors associated with disease recurrence and progression within 1 year are shown in Table 4, Table 5 and Table 6. In univariate analysis, a high-risk of NMIBC was associated with tumor recurrence (odds ratio (OR), 5.40; 95% confidence interval (CI), 1.12–26.04; *p* = 0.036) (Table 4). In addition, multivariate analysis identified old age (OR, 1.08; 95% CI, 1.01–1.15; *p* = 0.029) and low BMI (OR, 0.83; 95% CI, 0.70–0.99; *p* = 0.046) as significant factors associated with the progression of MIBC and UTUC within 1 year (Table 5). As shown in Table 6, *FGFR3* mutations may potentially affect bladder tumor recurrence in UTUC (OR, 11.00; 95% CI, 0.96–125.77; *p* = 0.054); however, the result was not statistically significant.

## 4. Discussion

UC is a heterogeneous malignancy and has a different clinical course depending on its histopathology and location [3,4,16]. In order to improve the assessment of the disease risk and prediction of the treatment response and prognosis, physicians have been focusing on advanced technologies such as genomic evaluation or artificial intelligence and gaining insights into a comprehensive cancer landscape [17]. Recently, the rapid development of NGS technology has allowed researchers to obtain comprehensive genetic information on cancer by leveraging genomic data based on NGS analysis [8]. Among the genomic biomarkers of UC, *FGFR3* is a potential biomarker for clinical decision making in disease diagnosis and management [9,10,12,18]. Therefore, in the present study, we evaluated the *FGFR3* mutation rate and its clinical significance in UC using NGS, and we found that *FGFR3* mutations were more prevalent in NMIBC and lower stage UC and associated with a high-risk of NMIBC. In addition, our findings suggest the potential of *FGFR3* as a biomarker for UC. 

FGFRs are highly conserved tyrosine kinase receptors and play essential roles in different cellular processes such as regulation of cell growth, proliferation, differentiation and death [19]. The *FGFR3* gene, which encodes one of the members of the FGFR family, has recently been found to be associated with UC oncogenesis, invasiveness and prognosis [20]. In addition, *FGFR3* mutations are one of the most common somatic mutations in UC, occurring in approximately 40–60% of cases of primary bladder and upper tract UC [10,21,22]. In the present study, 30.1% of 123 UC tumors had *FGFR3* mutations, which is relatively low compared with the percentage in other studies. This difference may be attributed to the varying location and stage of UC in the present study, unlike other studies. In particular, in the NMIBC group, the *FGFR3* mutation rate was 45.1%.

As mentioned above, the *FGFR3* mutation rate may be different according to the location and stage of UC, which is generally associated with lower disease grade and stage [10]. In several studies, *FGFR3* mutation rates were 49–84% in NMIBC compared with the rates (15–20%) in MIBC [10,11,21,23,24]. Most NMIBCs are characterized by papillary tumors, activating *FGFR3* mutations and genomic stability, in which *FGFR3* mutations play a role in inducing constitutive receptor activation that functions to promote proliferation via downstream activation of the extracellular signal-regulated kinases (ERKs) [25,26]. On the other hand, most MIBCs are characterized by non-papillary tumors, *TP53* and *RB1* inactivation and genomic instability [23,25]. In addition, *FGFR3* mutations are known to be mutually exclusive to some bladder cancer oncogenes, such as *TP53* and *RB1*, which are generally associated with MIBC [9]. Therefore, activating *FGFR3* mutations are less common in MIBC than in NMIBC. In the present study, the *FGFR3* mutation rates of the NMIBC and MIBC groups were 45.1% (23/51) and 22.7% (10/44), respectively, consistent with the findings of previous studies. 

In comparison with bladder cancer, UTUC is rare and accounts for only 5–10% of all UCs [5]. Consequently, studies on *FGFR3* mutations in UTUC have been uncommon. Nevertheless, several studies have reported *FGFR3* mutation rates in the range of 10–40% in UTUC [22,27,28,29,30]. In addition, *FGFR3* mutations in UTUC may be differentiated according to the disease stage, similar to bladder cancer. In particular, Sfakianos et al. and Lyle et al. reported *FGFR3* mutation rates of 30.4% and 40%, respectively, which were predominantly associated with non-muscle-invasive tumors [22,27]. In contrast, Springer et al. and Lee et al. reported *FGFR3* mutation rates of 21% and 13% in UTUC tissues, respectively, and the majority of UTUCs were categorized as muscle-invasive [29,30]. All UTUCs included in the present study were muscle-invasive, and the *FGFR3* mutation rate in the UTUC group was 14.3%, which is consistent with previous reports on *FGFR3* mutations in muscle-invasive UTUC.

As a prognostic biomarker of UC, *FGFR3* mutations are generally associated with a less aggressive tumor and favorable prognosis [10,11,21,24,31,32]. In previous studies on NMIBC, the *FGFR3* mutation rate was significantly higher in lower grade disease than in higher grade disease, which was associated with favorable outcomes [10,21,24]. In a study on the role of *FGFR3* mutations in primary T1 tumors, better progression-free survival was observed among patients with bladder cancer harboring *FGFR3* mutations [31]. In addition, studies on the molecular subtypes of bladder cancer revealed that *FGFR3* mutations may be associated with the luminal-papillary subtype, which itself is associated with a less aggressive phenotype and significantly longer overall survival compared with other subtypes [9,32]. Even in cases of MIBC, *FGFR3* mutations were associated with favorable pathologic features and longer disease-specific survival [11]. However, despite reports on the association of *FGFR3* mutations with favorable characteristics, there is also evidence suggesting that *FGFR3* mutations may be associated with less favorable outcomes in UC. Particularly in the context of tumor recurrence, *FGFR3* mutations may be strongly associated with a high-risk of recurrence in stage Ta tumors [21]. Although the underlying mechanism is unclear, bladder cancer development is thought to involve a tumoral epithelial-to-mesenchymal transition [33]. In the present study, *FGFR3* mutations were associated with lower stage UC but a high-risk of NMIBC. Taken together, the findings suggest that *FGFR3* may be one of the critical biomarkers of UC, and further studies will be needed to evaluate its clinical significance and apply it to actual clinical practice.

The present study is not without its limitations. First, this small-scale study only included patients from a single institution. In addition, the follow-up period of the present study is relatively short; we evaluated the effects on disease recurrence and progression within 1 year. Second, all UTUCs included in the present study were muscle-invasive, which may have a selection bias. Therefore, further studies with a longer follow-up period using more UC samples from multiple institutions are needed to clarify the precise role of *FGFR3* mutations. Third, our targeted sequencing approach for cancer sample tissues could be limited by biopsy bias and make it difficult to evaluate precise evolutionary relationships over time. Lastly, we did not consider the effect of adjuvant treatment. As adjuvant treatment can result in temporal changes in the genomic environment of UC, further studies on recurrent or progressed UC samples are needed to account for this aspect. 

Nevertheless, our results indicated that *FGFR3* may be an important biomarker of UC. Considering the results of this study, current strategies for the diagnosis of UC via conventional imaging and urine cytology may have a poor detection rate; thus, *FGFR3* mutation analysis should be considered for diagnosis and disease monitoring. In addition, although the mainstay of therapeutic approaches for the treatments of advanced or metastatic UC has so far been platinum-based chemotherapies, the development of *FGFR3*-targeting therapeutics (e.g., erdafitinib) will represent a milestone for the treatment of UC patients with *FGFR* mutation. Moreover, uncovering the complete mechanisms of *FGFR3*-targeting agent could provide advanced therapeutic strategies to increase efficacy, such as the combination with other immune checkpoint inhibitors or anti-neoplastic drugs. Lastly, although *FGFR3*-targeted therapy is performed in clinical practice for patients with MIBC, additional studies are needed to provide *FGFR3*-targeted therapy to patients with UTUC or NMIBC.

## 5. Conclusions

*FGFR3* mutations are one of the most prevalent somatic mutations in UC; thus, *FGFR3* may serve as a biomarker for patient selection, disease diagnosis and prognosis monitoring. In the present study, *FGFR3* mutations were more prevalent in NMIBC and lower stage UC and associated with a high-risk of NMIBC. In the future, additional studies to validate these results and evaluate clinical applications are needed.

## Figures and Tables

**Table 1:** Demographic characteristics of patients.

Variables	n = 123
Age (years)	71.39 ± 9.33
BMI (kg/m^2^)	24.10 ± 3.53
Sex	
Male	103 (83.7%)
Female	20 (16.3%)
Comorbidities	
Hypertension	70 (56.9%)
Diabetes mellitus	29 (23.6%)
NMIBC	51 (41.5%)
MIBC	44 (35.8%)
UTUC	28 (22.8%)
Stage	
0a	48 (39.0%)
1	3 (2.4%)
2	8 (6.5%)
3	39 (31.7%)
4	25 (20.3%)
*FGFR3* mutation	37 (30.1%)
Risk stratification for NMIBC (n = 51)	
Low	14 (27.4%)
Intermediate	21 (41.2%)
High	16 (31.4%)
Recurrence within 1 year (NMIBC, n = 51)	28 (54.9%)
Curative radical surgery (MIBC and UTUC, n = 72)	27 (39.7%)
Progression within 1 year (MIBC and UTUC, n = 72)	26 (36.1%)
Bladder tumor recurrence within 1 year (UTUC, n = 28)	4 (14.3%)

BMI: body mass index, NMIBC: non-muscle-invasive bladder cancer, MIBC: muscle-invasive bladder cancer, UTUC: upper tract urothelial carcinoma, FGFR: fibroblast growth factor receptor. Data are presented as the mean ± standard deviation or N (%).

**Table 2:** Comparisons of clinical features according to the type of UC.

Variable	NMIBC (n = 51)	MIBC (n = 44)	UTUC (n = 28)	*p* Value
Age (years)	71.08 ± 9.09	72.68 ± 10.01	69.93 ± 8.71	0.236 ^a^
BMI (kg/m^2^)	25.03 ± 3.32	23.20 ± 3.81	23.82 ± 3.12	0.036 ^b^
Sex				0.312 ^c^
Male	45 (88.2%)	37 (84.1%)	21 (75.0%)	
Female	6 (11.8%)	7 (15.9%)	7 (25.0%)	
Hypertension	29 (56.9%)	26 (59.1%)	15 (53.6%)	0.899 ^c^
Diabetes mellitus	14 (27.4%)	8 (18.2%)	7 (25.0%)	0.558 ^c^
Stage				<0.001 ^d^
0a	48 (94.1%)	0 (0.0%)	0 (0.0%)	
1	3 (5.9%)	0 (0.0%)	0 (0.0%)	
2	0 (0.0%)	3 (6.8%)	5 (17.9%)	
3	0 (0.0%)	30 (68.2%)	9 (32.1%)	
4	0 (0.0%)	11 (25.0%)	14 (50.0%)	
*FGFR3* mutation	23 (45.1%)	10 (22.7%)	4 (14.3%)	0.007 ^c^

BMI: body mass index, NMIBC: non-muscle-invasive bladder cancer, MIBC: muscle-invasive bladder cancer, UTUC: upper tract urothelial carcinoma, FGFR: fibroblast growth factor receptor. ^a^: Kruskal-Wallis rank test, ^b^: one-way ANOVA, ^c^: Pearson chi-square test, ^d^: Fisher’s exact test.

**Table 3:** Comparison of clinical features according to the presence of *FGFR3* mutations.

Variable	No (n = 86)	Yes (n = 37)	*p* Value
Age (years)	70.71 ± 9.64	72.97 ± 8.50	0.219 ^a^
BMI (kg/m^2^)	23.97 ± 3.50	24.41 ± 3.63	0.530 ^a^
Sex			0.291 ^b^
Male	74 (86.1%)	29 (78.4%)	
Female	12 (13.9%)	8 (21.6%)	
Hypertension	47 (54.7%)	23 (62.2%)	0.440 ^b^
Diabetes mellitus	21 (24.4%)	8 (21.6%)	0.738 ^b^
Stage			0.019 ^c^
0a	26 (30.2%)	22 (59.5%)	
1	2 (2.3%)	1 (2.7%)	
2	7 (8.1%)	1 (2.7%)	
3	29 (33.7%)	10 (27.0%)	
4	22 (25.6%)	3 (8.1%)	
Risk stratification for NMIBC (n = 51)			<0.001 ^c^
Low	13 (46.4%)	1 (4.4%)	
Intermediate	10 (35.7%)	11 (47.8%)	
High	5 (17.9%)	11 (47.8%)	
Recurrence within 1 year (NMIBC, n = 51)	13 (46.4%)	15 (65.2%)	0.180 ^b^
Progression within 1 year (MIBC and UTUC, n = 72)	18 (31.0%)	8 (57.1%)	0.068 ^b^
Bladder tumor recurrence within 1 year (UTUC, n = 28)	2 (8.3%)	2 (50.0%)	0.086 ^b^

BMI: body mass index, NMIBC: non-muscle-invasive bladder cancer, MIBC: muscle-invasive bladder cancer, UTUC: upper tract urothelial carcinoma, FGFR: fibroblast growth factor receptor. ^a^: Student’s *t*-test, ^b^: Pearson chi-square test, ^c^: Fisher’s exact test.

**Table 4:** Clinical factors associated with the recurrence of NMIBC within 1 year.

Variables	Univariate Analysis		Multivariate Analysis	
	Odds Ratio (95% CI)	*p* Value	Odds Ratio (95% CI)	*p* Value
Age (years)	1.03 (0.97–1.10)	0.340	Not applicable	
BMI (kg/m^2^)	0.99 (0.84–1.17)	0.893		
Sex				
Male	Reference			
Female	0.80 (0.15–4.40)	0.798		
Hypertension	0.53 (0.17–1.66)	0.277		
Diabetes mellitus	0.51 (0.15–1.78)	0.291		
Stage				
0a	Reference			
1	1.69 (0.14–19.94)	0.676		
Risk stratification				
Low	Reference			
Intermediate	1.98 (0.49–7.94)	0.335		
High	5.40 (1.12–26.04)	0.036		
*FGFR3* mutation	2.16 (0.70–6.73)	0.183		

BMI: body mass index, NMIBC: non-muscle-invasive bladder cancer, FGFR: fibroblast growth factor receptor.

**Table 5:** Clinical factors associated with the progression of MIBC and UTUC within 1 year.

Variables	Univariate Analysis		Multivariate Analysis	
	Odds Ratio (95% CI)	*p* Value	Odds Ratio (95% CI)	*p* Value
Age (years)	1.10 (1.03–1.17)	0.007	1.08 (1.01–1.15)	0.029
BMI (kg/m^2^)	0.85 (0.73–0.99)	0.032	0.83 (0.70–0.99)	0.046
Sex				
Male	Reference			
Female	1.43 (0.43–4.68)	0.559		
Hypertension	2.96 (1.04–8.39)	0.041	3.00 (0.92–9.81)	0.069
Diabetes mellitus	0.58 (0.16–2.04)	0.396		
Stage				
2	Reference			
3	5.41 (0.61–48.27)	0.131		
4	3.29 (0.34–31.49)	0.301		
UTUC	0.33 (0.11–0.96)	0.043	0.37 (0.11–1.21)	0.100
Curative radical surgery	0.35 (0.12–1.05)	0.061		
*FGFR3* mutation	2.96 (0.90–9.80)	0.075		

BMI: body mass index, MIBC: muscle-invasive bladder cancer, UTUC: upper tract urothelial carcinoma, FGFR: fibroblast growth factor receptor.

**Table 6:** Clinical factors associated with bladder tumor recurrence in UTUC within 1 year.

Variables	Univariate Analysis		Multivariate Analysis	
	Odds Ratio (95% CI)	*p* Value	Odds Ratio (95% CI)	*p* Value
Age (years)	1.08 (0.92–1.26)	0.340	Not applicable	
BMI (kg/m^2^)	0.81 (0.54–1.23)	0.325		
Sex				
Male	Reference			
Female	1.00 (0.09–11.52)	1.000		
Hypertension	3.00 (0.27–33.08)	0.370		
Diabetes mellitus	0.33 (0.03–3.34)	0.350		
Stage				
2	Reference			
3	2.00 (0.15–26.73)	0.600		
4	1.60 (0.13–19.09)	0.710		
*FGFR3* mutation	11.00 (0.96–125.77)	0.054		

BMI: body mass index, UTUC: upper tract urothelial carcinoma, FGFR: fibroblast growth factor receptor.

## Data Availability

The data presented in this study are available in this article.
